# A National Pension Fund for Hospital Officials and Nurses

**Published:** 1887-05-07

**Authors:** 


### A National Pension Fund for Hospital Officials and Nurses.

### The Views of Officials, Sisters, Nurses, and others.

**By a Monthly Nurse and Trained Midwife.**

I am much interested in the discussion in The Hospital, on a National Pension Fund for Nurses, which is a want sadly hitherto overlooked, and I venture to make a suggestion, that such a fund might perhaps be started on the plan of "The Servants' Benevolent Fund," Sackville Street, and should certainly include midwives and private nurses. I should be very glad to see the "Rules" of the present fund, and happy to join such a fund, for although having worked now twenty years as monthly nurse and trained midwife, I have never yet joined or knew of such a fund having been started. I will not do more now than state the deep interest I shall take in the proposal, and give it all the help in my power.

*** The Servants' Benevolent Fund is a philanthropic institution in no way suited to the present purpose. Self-help and independence must be the watchword of the National Pension Fund.—[Ed. T. H.]

**By Miss M. J. Plenney, Matron-Nurse, Cottage Hospital, Totnes.**

I am very pleased to see that an effort is being made to establish an Annuity Fund for nurses and hospital officials. Enough, I think, has been written to show that it would be an excellent and useful thing. I sincerely hope it may be established this Jubilee year. I shall be pleased to subscribe, and I feel sure many others would if they knew and understood about it. It has been suggested that a small poster be printed and sent to the matron of each hospital, setting forth the nature of the scheme. She might, perhaps, kindly ascertain how many of the staff would be willing to subscribe, and report to the honorary secretary with any suggestions or remarks. Many nurses (especially the younger ones) may not have the courage to write.

---

Any hospital officials or nurses willing to co-operate in the establishment of this fund, should promptly send their name and address to Mr. Howard J Collins, Secretary, The Hospitals Association, Norfolk House, Norfolk Street, W.C.

